# Pyridine-2,3-diamine

**DOI:** 10.1107/S1600536811029412

**Published:** 2011-07-30

**Authors:** Richard Betz, Thomas Gerber, Eric Hosten, Henk Schalekamp

**Affiliations:** a Nelson Mandela Metropolitan University, Summerstrand Campus, Department of Chemistry, University Way, Summerstrand, PO Box 77000, Port Elizabeth 6031, South Africa

## Abstract

The mol­ecule of the title pyridine derivative, C_5_H_7_N_3_, shows approximately non-crystallographic *C*_s_ symmetry. Intra­cyclic angles cover the range 117.50 (14)–123.03 (15)°. In the crystal, N—H⋯N hydrogen bonds connect mol­ecules into a three-dimensional network. The closest inter­centroid distance between two π-systems occurs with the *c*-axis repeat at 3.9064 (12) Å.

## Related literature

For the crystal structure of the dihydro­chloride of the title compound, see: Hemamalini & Fun (2010 ▶). For the crystal structures of Zn complexes of the title compound, see: de Cires-Mejias *et al.* (2004 ▶). For graph-set analysis of hydrogen bonds, see: Etter *et al.* (1990 ▶); Bernstein *et al.* (1995 ▶).
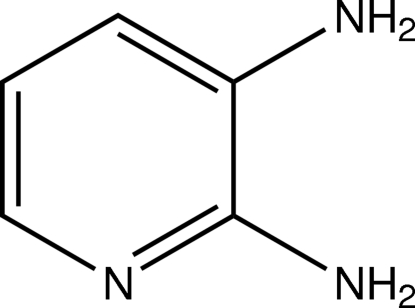


## Experimental

### 

#### Crystal data

C_5_H_7_N_3_
*M* *_r_* = 109.14
Tetragonal, 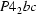

*a* = 16.4670 (3) Å
*c* = 3.9064 (12) Å
*V* = 1059.3 (3) Å^3^
*Z* = 8
Mo *K*α radiation
μ = 0.09 mm^−1^
*T* = 200 K
0.48 × 0.16 × 0.11 mm

#### Data collection

Bruker APEXII CCD diffractometer
9864 measured reflections
754 independent reflections
706 reflections with *I* > 2σ(*I*)
*R*_int_ = 0.047

#### Refinement

*R*[*F* ^2^ > 2σ(*F* ^2^)] = 0.036
*wR*(*F* ^2^) = 0.085
*S* = 1.10
754 reflections
89 parameters
1 restraint
H atoms treated by a mixture of independent and constrained refinement
Δρ_max_ = 0.23 e Å^−3^
Δρ_min_ = −0.17 e Å^−3^

### 

Data collection: *APEX2* (Bruker, 2010 ▶); cell refinement: *SAINT* (Bruker, 2010 ▶); data reduction: *SAINT*; program(s) used to solve structure: *SHELXS97* (Sheldrick, 2008 ▶); program(s) used to refine structure: *SHELXL97* (Sheldrick, 2008 ▶); molecular graphics: *ORTEP-3* (Farrugia, 1997 ▶) and *Mercury* (Macrae *et al.*, 2008 ▶); software used to prepare material for publication: *SHELXL97* and *PLATON* (Spek, 2009 ▶).

## Supplementary Material

Crystal structure: contains datablock(s) I, global. DOI: 10.1107/S1600536811029412/om2449sup1.cif

Supplementary material file. DOI: 10.1107/S1600536811029412/om2449Isup2.cdx

Structure factors: contains datablock(s) I. DOI: 10.1107/S1600536811029412/om2449Isup3.hkl

Supplementary material file. DOI: 10.1107/S1600536811029412/om2449Isup4.cml

Additional supplementary materials:  crystallographic information; 3D view; checkCIF report

## Figures and Tables

**Table 1:** Hydrogen-bond geometry (Å, °)

*D*—H⋯*A*	*D*—H	H⋯*A*	*D*⋯*A*	*D*—H⋯*A*
N2—H21⋯N1^i^	0.87 (2)	2.32 (2)	3.153 (2)	161.2 (19)
N2—H22⋯N2^ii^	0.85 (2)	2.58 (2)	3.4369 (16)	175.9 (18)
N3—H31⋯N1^iii^	0.86 (2)	2.32 (2)	3.115 (2)	156 (2)
N3—H32⋯N3^iv^	0.89 (3)	2.47 (2)	3.359 (2)	175 (2)

Symmetry codes: (i) 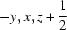; (ii) 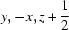; (iii) 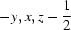; (iv) 
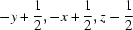
.

**Fig. 1.**
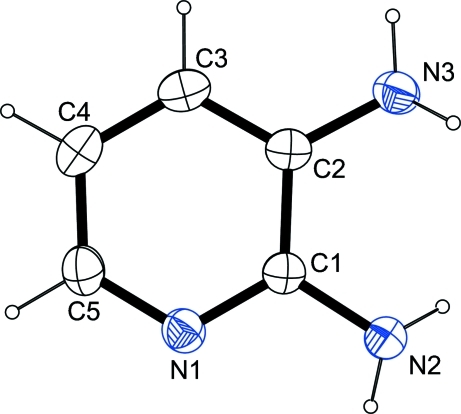
The molecular structure of the title compound, with atom labels and anisotropic displacement ellipsoids (drawn at 50% probability level).

**Fig. 2.**
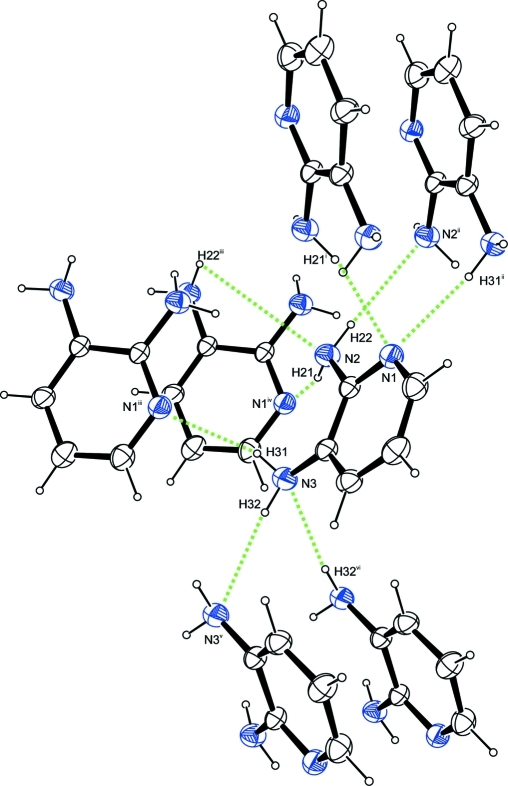
Intermolecular contacts, viewed approximately along [0 0 1]. Symmetry operators: iy, -x, z - 1/2; iiy, -x, z + 1/2; iii -y, x, z - 1/2; iv -y, x, z + 1/2; v -y + 1/2, -x + 1/2, z - 1/2; vi -y + 1/2, -x + 1/2, z + 1/2.

**Fig. 3.**
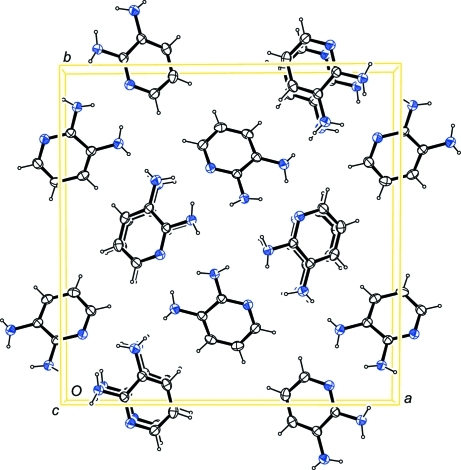
Molecular packing of the title compound, viewed along [0 0 - 1] (anisotropic displacement ellipsoids drawn at 50% probability level).

## supplementary crystallographic information

### Comment

Chelate ligands have found widespread use in coordination chemistry due to the enhanced thermodynamic stability of resultant coordination compounds in relation to coordination compounds exclusively applying comparable monodentate ligands. Combining identical donor atoms in different states of hybridization, a molecular set-up to accomodate a large variety of metal centers of variable Lewis acidity is at hand. In this aspect, the title compound seemed interesting due to its use as strictly neutral or – depending on the pH value – as anionic or cationic ligand. Furthermore, thanks to the presence of three possible donor atoms, the title compound might serve as a building block in the formation of metal-organic framework structures. For the title compound, two zinc-supported polymers have been reported whose crystal structure analysis shows the absence of chelate-type building motifs (de Cires-Mejias *et al.*, 2004). At the beginning of a more comprehensive study to elucidate the formation of coordination polymers exclusively featuring nitrogen-containing ligands, we determined the structure of the title compound to enable comparative studies of metrical parameters in envisioned coordination compounds. Information about the molecular and crystal structure of the dihydrochloride of the title compound is apparent in the literature (Hemamalini & Fun, 2010).

Intracyclic angles cover a range of 117.50 (14)–123.03 (15) ° with the smallest angle found on the carbon atom bearing the amino group in *meta* position to the intracyclic nitrogen atom and the biggest angle found on the carbon atom bearing a hydrogen atom in *ortho* position to the intracyclic nitrogen atom. Apart from the hydrogen atoms of the amino groups which point to opposite sides of the plane defined the aromatic system, all atoms are essentially residing in one common plane (r.m.s. deviation of all fitted non-hydrogen atoms = 0.0152 Å). The amino groups are not planar, the least-squares planes defined by the NH_2_ groups subtend angles of 40.2 (2) ° and 79.5 (2) ° with the least-squares plane defined by the atoms of the heterocycle (Fig. 1).

The crystal structure of the title compound is marked by a hydrogen bonding system involving all hydrogen atoms of both amino groups as donors and the intracyclic as well as the exocyclic nitrogen atoms as acceptors. The intracyclic nitrogen atom serves as a twofold acceptor for one of the hydrogen atoms of each of the two different amino groups. The remaining hydrogen atom on each amino group gives rise to a cooperative chain of hydrogen bonds, respectively. The latter ones are antidromic. In terms of graph-set analysis (Etter *et al.*, 1990; Bernstein *et al.*, 1995), the descriptor for this hydrogen bonding system on the unitary level is *C*^1^_1_(2)*C*^1^_1_(2)*C*^1^_1_(4)*C*^1^_1_(5). In total, the molecules are connected to a three-dimensional network (Fig. 2). The closest intercentroid distance between two aromatic systems follows the c-axis repeat at 3.9064 (12) Å.

The packing of the title compound is shown in Figure 3.

### Experimental

The compound was obtained commercially (Aldrich). Crystals suitable for the X-ray diffraction study were taken directly from the provided compound.

### Refinement

Carbon-bound H atoms were placed in calculated positions (C—H 0.95 Å) and were included in the refinement in the riding model approximation, with *U*(H) set to 1.2*U*_eq_(C). The H atoms of the amine groups were located on a difference Fourier map and refined with individual thermal parameters. Due to the absence of a strong anomalous scatterer, the Flack parameter is meaningless. Thus, Friedel opposites (2407 pairs) have been merged and the item was removed from the CIF.

### Crystal data

**Table:**

C_5_H_7_N_3_	*D*_x_ = 1.369 Mg m^−^^3^
*M**_r_* = 109.14	Mo *K*α radiation, λ = 0.71073 Å
Tetragonal, *P*4_2_*b**c*	Cell parameters from 5917 reflections
Hall symbol: P 4c -2ab	θ = 2.5–28.3°
*a* = 16.4670 (3) Å	µ = 0.09 mm^−^^1^
*c* = 3.9064 (12) Å	*T* = 200 K
*V* = 1059.3 (3) Å^3^	Needle, brown
*Z* = 8	0.48 × 0.16 × 0.11 mm
*F*(000) = 464	

### Data collection

**Table:**

Bruker APEXII CCD diffractometer	706 reflections with *I* > 2σ(*I*)
Radiation source: fine-focus sealed tube	*R*_int_ = 0.047
graphite	θ_max_ = 28.3°, θ_min_ = 1.8°
φ and ω scans	*h* = −21→20
9864 measured reflections	*k* = −21→21
754 independent reflections	*l* = −5→5

### Refinement

**Table:**

Refinement on *F*^2^	Primary atom site location: structure-invariant direct methods
Least-squares matrix: full	Secondary atom site location: difference Fourier map
*R*[*F*^2^ > 2σ(*F*^2^)] = 0.036	Hydrogen site location: inferred from neighbouring sites
*wR*(*F*^2^) = 0.085	H atoms treated by a mixture of independent and constrained refinement
*S* = 1.10	*w* = 1/[σ^2^(*F*_o_^2^) + (0.0428*P*)^2^ + 0.2489*P*] where *P* = (*F*_o_^2^ + 2*F*_c_^2^)/3
754 reflections	(Δ/σ)_max_ < 0.001
89 parameters	Δρ_max_ = 0.23 e Å^−^^3^
1 restraint	Δρ_min_ = −0.17 e Å^−^^3^

### Fractional atomic coordinates and isotropic or equivalent isotropic displacement parameters (Å^2^)

**Table:**

	*x*	*y*	*z*	*U*_iso_*/*U*_eq_	
N1	0.20596 (8)	−0.05610 (8)	0.9243 (5)	0.0268 (3)	
N2	0.11296 (8)	0.04458 (9)	1.0621 (5)	0.0271 (3)	
H21	0.1094 (13)	0.0921 (14)	1.156 (8)	0.040 (6)*	
H22	0.0934 (12)	0.0063 (14)	1.184 (7)	0.037 (6)*	
N3	0.21809 (9)	0.16454 (9)	0.7974 (5)	0.0302 (4)	
H31	0.1677 (13)	0.1730 (12)	0.757 (7)	0.033 (5)*	
H32	0.2471 (12)	0.1946 (12)	0.652 (8)	0.038 (6)*	
C1	0.18758 (9)	0.02266 (9)	0.9286 (5)	0.0225 (4)	
C2	0.23965 (9)	0.08211 (9)	0.7834 (6)	0.0240 (3)	
C3	0.31167 (10)	0.05570 (10)	0.6425 (5)	0.0283 (4)	
H3	0.3478	0.0938	0.5417	0.034*	
C4	0.33173 (10)	−0.02631 (11)	0.6469 (6)	0.0307 (4)	
H4	0.3819	−0.0449	0.5556	0.037*	
C5	0.27702 (10)	−0.07971 (10)	0.7870 (6)	0.0300 (4)	
H5	0.2900	−0.1359	0.7869	0.036*	

### Atomic displacement parameters (Å^2^)

**Table:**

	*U*^11^	*U*^22^	*U*^33^	*U*^12^	*U*^13^	*U*^23^
N1	0.0256 (7)	0.0250 (7)	0.0298 (8)	0.0002 (5)	−0.0024 (7)	0.0000 (7)
N2	0.0250 (7)	0.0249 (7)	0.0315 (8)	−0.0003 (5)	0.0025 (6)	−0.0013 (7)
N3	0.0276 (7)	0.0256 (7)	0.0374 (9)	−0.0023 (5)	−0.0009 (8)	0.0039 (8)
C1	0.0214 (7)	0.0252 (7)	0.0209 (8)	−0.0013 (5)	−0.0042 (7)	−0.0005 (7)
C2	0.0238 (7)	0.0267 (7)	0.0217 (7)	−0.0032 (5)	−0.0040 (7)	0.0003 (8)
C3	0.0261 (8)	0.0357 (8)	0.0231 (8)	−0.0065 (6)	−0.0014 (8)	0.0009 (8)
C4	0.0242 (7)	0.0411 (9)	0.0268 (9)	0.0024 (6)	0.0012 (8)	−0.0044 (9)
C5	0.0288 (8)	0.0278 (8)	0.0334 (9)	0.0045 (6)	−0.0023 (10)	−0.0021 (9)

### Geometric parameters (Å, °)

**Table:**

N1—C1	1.332 (2)	C1—C2	1.420 (2)
N1—C5	1.345 (2)	C2—C3	1.378 (2)
N2—C1	1.383 (2)	C3—C4	1.390 (2)
N2—H21	0.87 (2)	C3—H3	0.9500
N2—H22	0.85 (2)	C4—C5	1.373 (3)
N3—C2	1.404 (2)	C4—H4	0.9500
N3—H31	0.86 (2)	C5—H5	0.9500
N3—H32	0.89 (3)		
			
C1—N1—C5	118.97 (15)	C3—C2—C1	117.50 (14)
C1—N2—H21	117.1 (15)	N3—C2—C1	119.89 (16)
C1—N2—H22	110.7 (14)	C2—C3—C4	120.41 (16)
H21—N2—H22	114 (2)	C2—C3—H3	119.8
C2—N3—H31	113.3 (13)	C4—C3—H3	119.8
C2—N3—H32	112.2 (14)	C5—C4—C3	118.11 (16)
H31—N3—H32	108 (2)	C5—C4—H4	120.9
N1—C1—N2	117.44 (15)	C3—C4—H4	120.9
N1—C1—C2	121.95 (15)	N1—C5—C4	123.03 (15)
N2—C1—C2	120.49 (14)	N1—C5—H5	118.5
C3—C2—N3	122.57 (16)	C4—C5—H5	118.5
			
C5—N1—C1—N2	178.05 (17)	N3—C2—C3—C4	177.43 (19)
C5—N1—C1—C2	1.9 (3)	C1—C2—C3—C4	−0.4 (3)
N1—C1—C2—C3	−1.4 (3)	C2—C3—C4—C5	1.6 (3)
N2—C1—C2—C3	−177.42 (18)	C1—N1—C5—C4	−0.6 (3)
N1—C1—C2—N3	−179.3 (2)	C3—C4—C5—N1	−1.1 (3)
N2—C1—C2—N3	4.7 (3)		

### Hydrogen-bond geometry (Å, °)

**Table:**

*D*—H···*A*	*D*—H	H···*A*	*D*···*A*	*D*—H···*A*
N2—H21···N1^i^	0.87 (2)	2.32 (2)	3.153 (2)	161.2 (19)
N2—H22···N2^ii^	0.85 (2)	2.58 (2)	3.4369 (16)	175.9 (18)
N3—H31···N1^iii^	0.86 (2)	2.32 (2)	3.115 (2)	156 (2)
N3—H32···N3^iv^	0.89 (3)	2.47 (2)	3.359 (2)	175 (2)

Symmetry codes: (i) −*y*, *x*, *z*+1/2; (ii) *y*, −*x*, *z*+1/2; (iii) −*y*, *x*, *z*−1/2; (iv) −*y*+1/2, −*x*+1/2, *z*−1/2.
